# Case Report: Diagnostic challenges in VEXAS syndrome with novel ultrastructural lung findings: IgG4-RD and vasculitis as relevant differential diagnoses

**DOI:** 10.3389/fimmu.2025.1742328

**Published:** 2026-01-21

**Authors:** Peter Etzel, David Lang, Walter Stoiber, Astrid Obermayer, Gregor Öberseder, Bernd Lamprecht

**Affiliations:** 1 Johannes Kepler University Linz, Kepler University Hospital, Department of Pulmonology, Linz, Austria; 2 Department of Environment and Biodiversity – EM Core Facility, University of Salzburg, Salzberg, Austria; 3 Kepler University Hospital, Department of Rheumatology, Linz, Austria

**Keywords:** organizing pneumonia, ruxolitinib, transbronchial lung cryobiopsy, transmission electron microscopy (TEM), UBA1 mutation

## Abstract

VEXAS syndrome is a rare, adult-onset autoinflammatory disorder caused by somatic mutations in the UBA1 gene. Patients may present with symptoms similar to IgG4-related disease (IgG4-RD) or systemic vasculitis. We report the case of a 70-year-old man who presented with periorbital swelling, fever, and elevated serum IgG4. However, a biopsy of the lacrimal gland did not show histological evidence of IgG4-RD. Consecutively, the patient developed progressive pulmonary infiltrations, bicytopenia and leukocytoclastic vasculitis. Chest-CT showed organizing pneumonia, which was histologically proven by transbronchial lung cryobiopsy (TBLC), again excluding IgG4-RD. PET/CT revealed hypermetabolic bone marrow and bone marrow aspiration biopsy showed vacuolization of granulocytic precursor cells. Finally, genetic testing for UBA1 mutation confirmed the diagnosis of VEXAS syndrome. Treatment with ruxolitinib in addition to steroids, led to temporary stabilization but long-term prognosis was unfavorable. This case highlights the importance of considering VEXAS syndrome a relevant differential diagnosis of vasculitis and IgG4-RD in men. Furthermore, we present valuable insights into the pathophysiology of VEXAS through transmission electron microscopy (TEM) of TBLC samples.

## 1 Introduction

VEXAS (Vacuoles, E1 enzyme, X-linked, Autoinflammatory, Somatic) syndrome is an autoinflammatory disease due to a somatic mutation in the UBA1 gene, occurring exclusively in adults (1). It is characterized by systemic inflammation, hematological abnormalities and often treatment-refractory clinical symptoms (2) including fever, skin lesions, chondritis, pulmonary infiltrates and macrocytic anemia, that may initially mimic other rheumatologic or hematologic diseases (1–5). If the disease relapses after initial corticosteroid therapy or if the necessary dose remains above 10 mg/day, additional corticosteroid-sparing therapies are recommended. Options listed in the American College of Rheumatology (ACR) guidelines include anti-IL6 therapy with (e.g. tocilizumab), JAK inhibitors (such as ruxolitinib), or anti-IL1 drugs like canakinumab (6).

In addition to ruxolitinib, a recent comprehensive review on treatment options also reported successful use of azacitidine and IL-6 inhibitors (7, 8), which are also included in the ACR guidelines. However, the prognosis for VEXAS syndrome remains unfavorable due to often-progressive myelodysplastic changes and complications such as thrombosis or infections (5, 9).

UBA1 mutations lead to a defective ubiquitination, resulting in protein accumulation and activation of inflammatory cytokines (IL-6, TNF-a and IFN-y) (1). Different UBA1 mutations are associated with variations in organ involvement and prognosis (10, 11). In a retrospective cohort study, out of 45 VEXAS patients with lung involvement, a total of 6 underwent lung biopsies (3 bronchoscopic and 3 surgical), which showed organizing pneumonia, lymphoid interstitial pneumonia, wild-type transthyretin amyloidosis (ATTR), interstitial fibrosis, atypical T-cell population, and normal parenchyma (12). Further examination of subcellular structures using transmission electron microscopy has not yet been described in literature and could provide additional insights into the pathophysiology of VEXAS.

IgG4-related disease (IgG4-RD) is a chronic fibroinflammatory disease characterized by tissue swelling, infiltration with IgG4-positive plasma cells and frequently elevated IgG4 plasma levels. Due to clinical and radiologic overlaps with other immune-mediated or neoplastic diseases, IgG4-RD is often misdiagnosed, particularly in the absence of typical histopathological features. Vasculitis is commonly associated with recurrent fever, elevated CRP levels, and involvement of multiple organs, which can mimic the clinical presentation of VEXAS syndrome (2, 13, 14).

## 2 Case description and clinical findings

A 70-year old man presented to a rheumatology unit with left-sided ptosis and periorbital soft tissue swelling. His medical history included peripheral arterial disease, arterial hypertension, diabetes mellitus type 2, and coronary artery disease with a previous myocardial infarction. Initial laboratory tests showed a mildly elevated C-reactive protein (CRP) level (2.6 mg/dL), and a white blood cell count of 6.6 G/L (reference 4.00-10.00 G/L). The patient exhibited normochromic, normocytic anemia with a hemoglobin level of 8.2 g/dL (reference 13.0-17.5 g/dL), and a normal platelet count. Further autoimmune marker testing showed negative results for: Anti-myeloperoxidase (Anti-MPO), Anti-proteinase 3 (Anti-PR3), Anti-neutrophil cytoplasmic antibodies (ANCAs), antinuclear antibodies (ANA), Anti-Ro (SSA), Anti-La (SSB). Serum protein electrophoresis (SPEP) was normal. Elevated serum IgG4 levels (total IgG not determined) were observed, suggesting IgG4-related disease as a differential diagnosis.

An excisional biopsy of the lacrimal gland, however, failed to demonstrate the typical histopathological features of IgG4-RD and also showed no evidence of any other specific disease. The patient presented petechial lesions on both lower legs, and the skin biopsy suggested leukocytoclastic vasculitis. Empirical treatment with intravenous clindamycin, metronidazole and high-dose corticosteroids (prednisolone 75 mg/day) was initiated, leading to clinical improvement and decrease in inflammatory markers.

**Figure 1:**
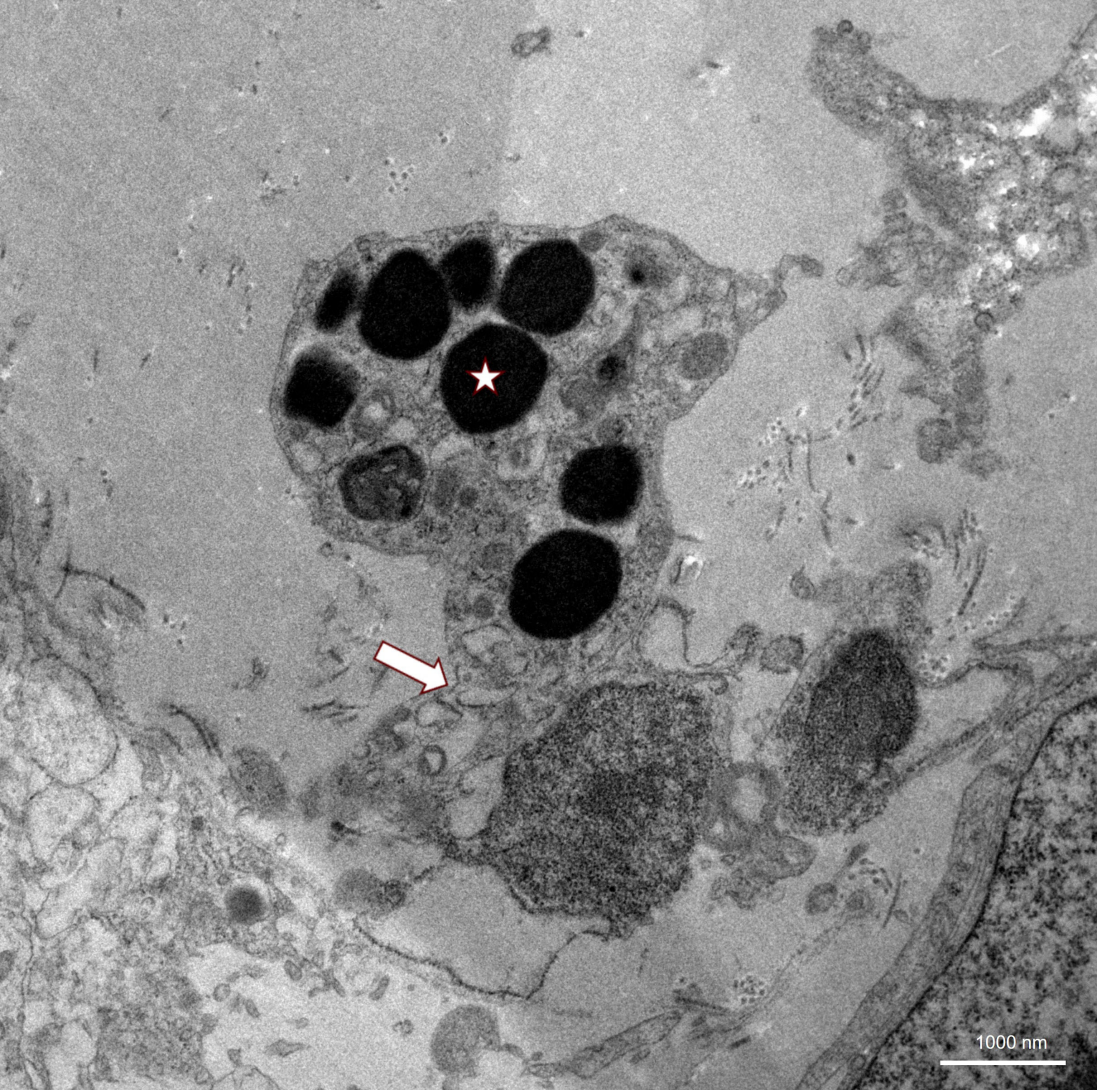
Ultrastructure of alveolar wall (arrow) in TBLC as seen in TEM, showing a pneumocyte with unclear electron-dense inclusions that could be interpreted as phagosomes (asterisk).

**Figure 2:**
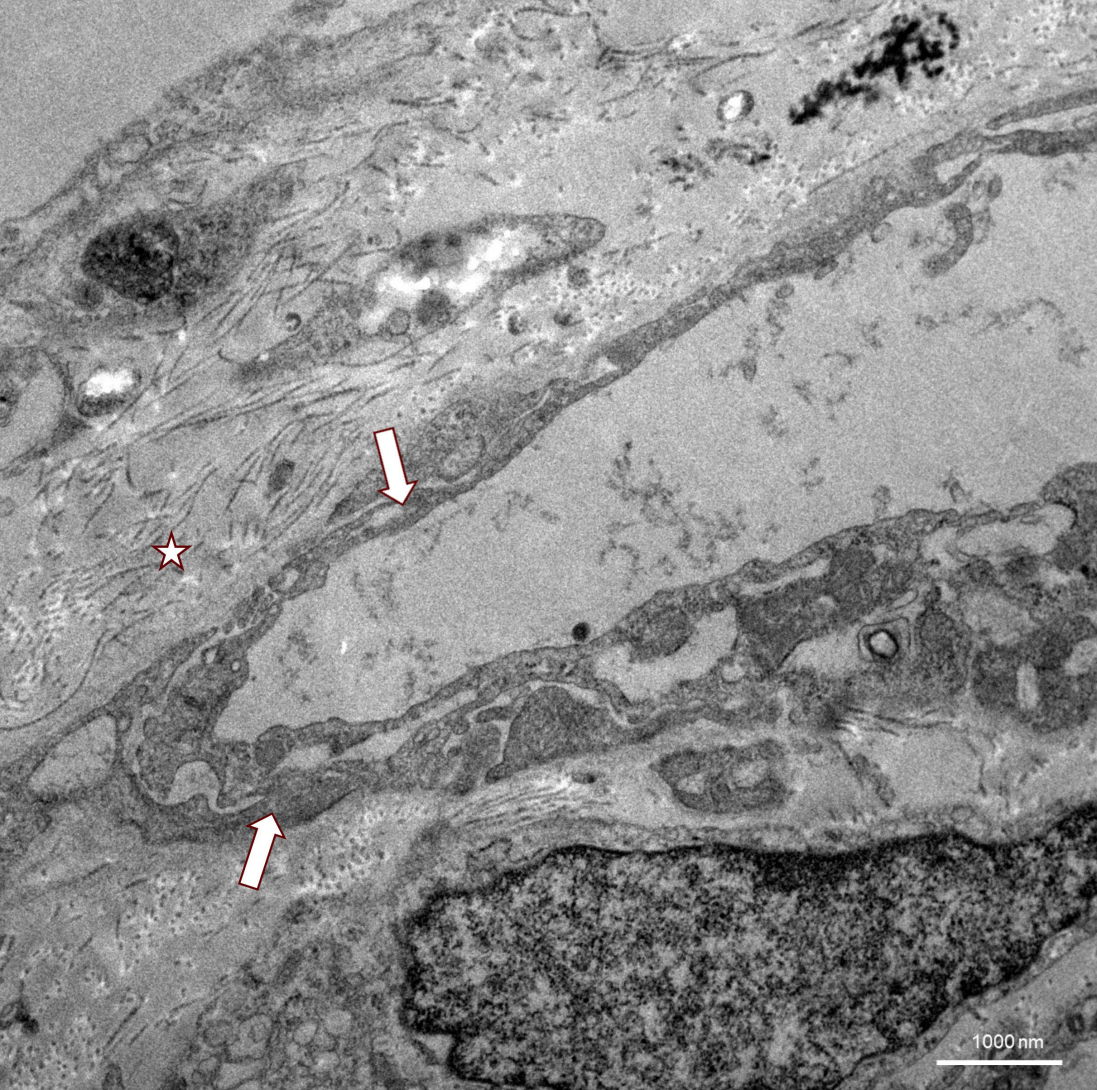
TEM of TBLC showing a massively thickened basement membrane with abundant collagen (asterisk) underneath a capillary with an endothelium of partly stratified appearance (arrows).

**Figure 3:**
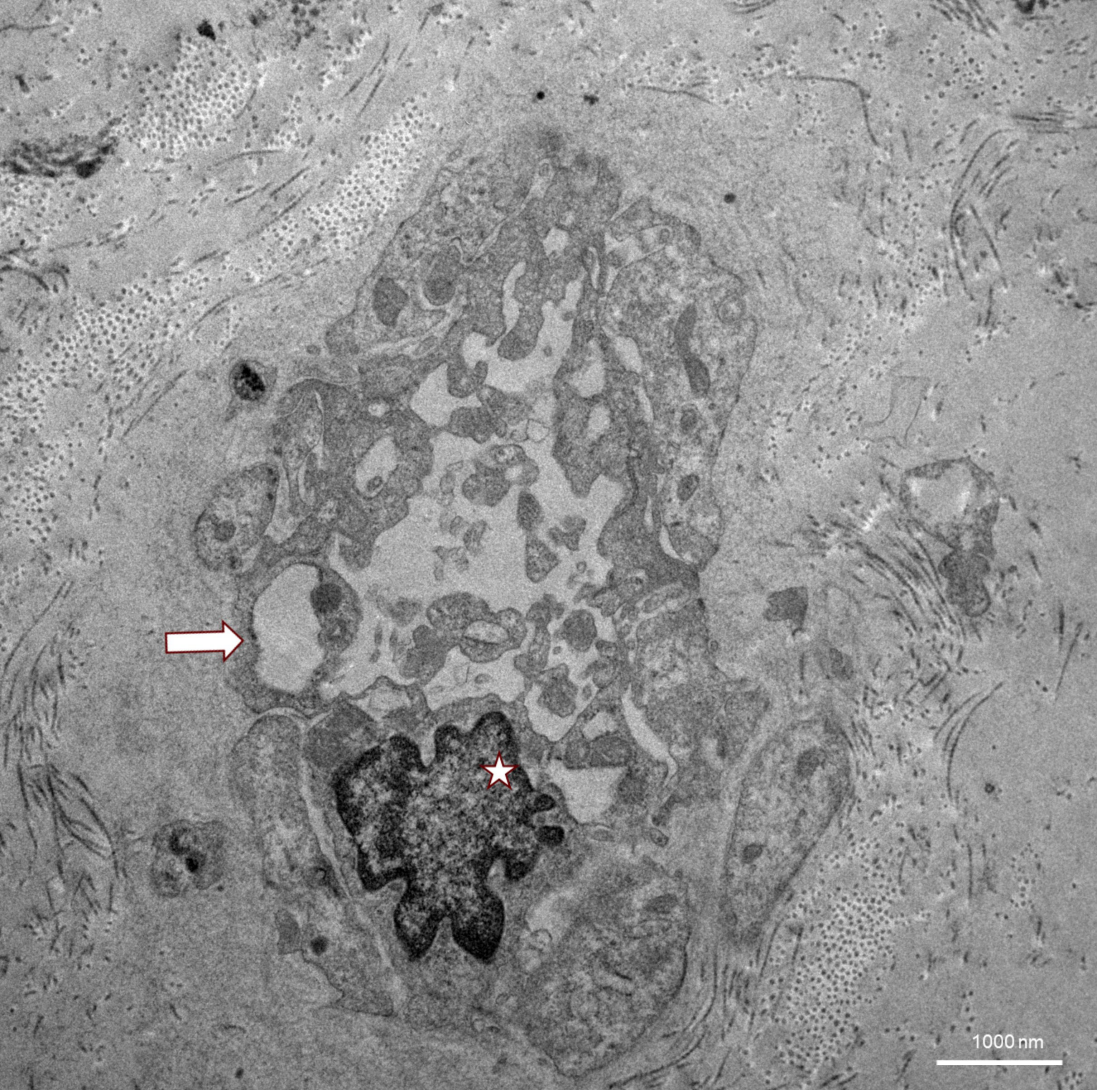
TEM image of lung tissue from a TBLC. Capillaries (arrow) may be almost completely blocked by pseudopodia. The structure marked with an asterisk is most likely an endothelial cell nucleus with aberrant indentations probably resulting from shear stress.

**Figure 4:**
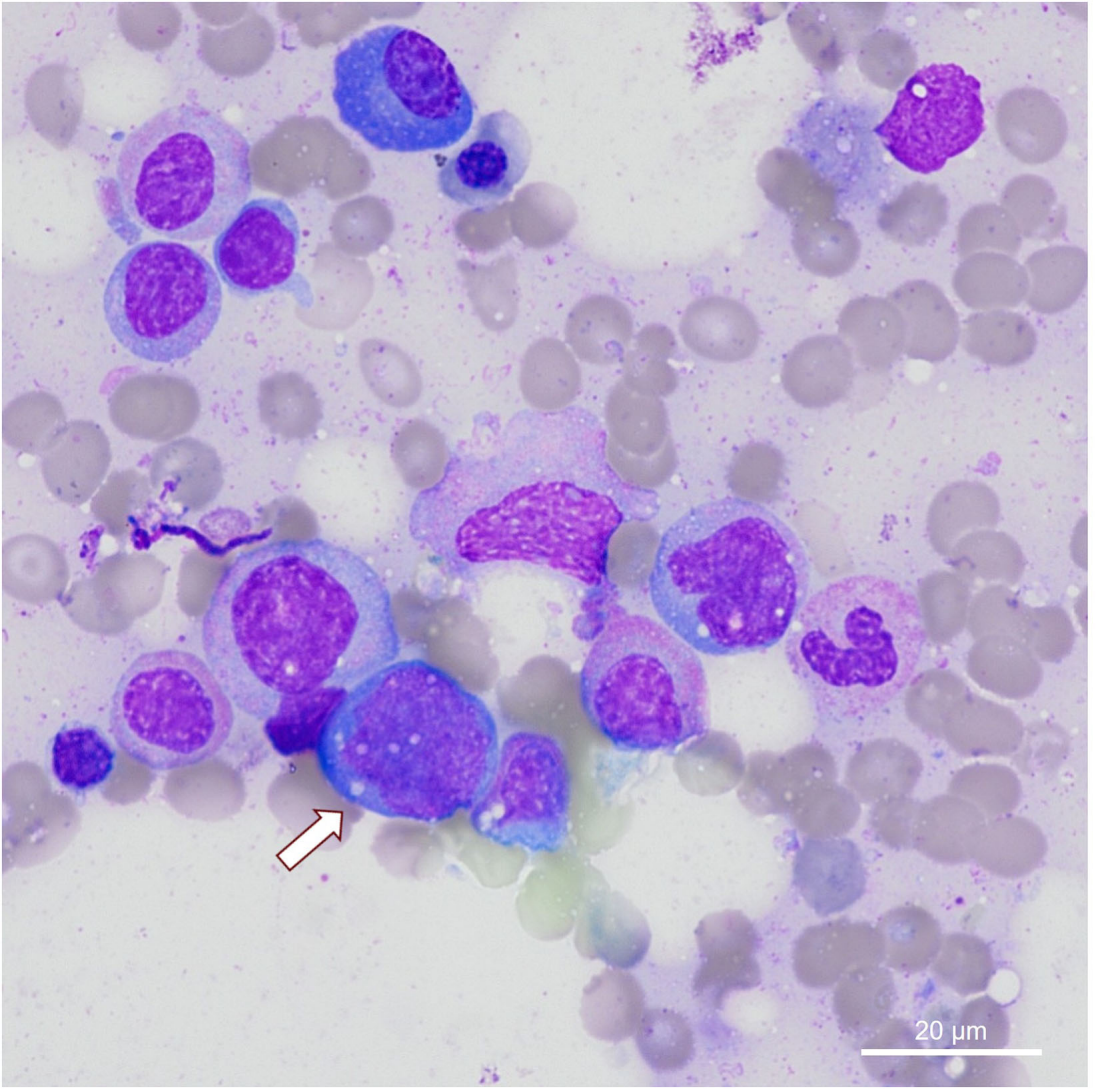
Bone marrow biopsy with detection of vacuolization (arrow) in granulocytic precursor cells in hematoxylin-eosin (HE) staining.

Two months later, the patient reported dry cough with absence of periorbital swelling during corticosteroid tapering. Chest CT revealed progressive bilateral basal infiltrates. A PET/CT scan performed due to persistent anemia and recurrent fever demonstrated diffuse hypermetabolic activity in the bone marrow with no other marked organ-specific uptake. Concomitant CT showed findings consistent with organizing pneumonia, and hepatosplenomegaly was described. Due to the progressive pulmonary findings and with IgG4-RD still being a primary differential diagnosis, bronchoscopy with TBLC was performed. Histologically, there was again no evidence of IgG4-RD, but findings were consistent with organizing pneumonia. The patient was enrolled into an observational trial focusing on TEM imaging of lung cryobiopsy samples of interstitial lung disease (ILD) patients, which enabled thorough investigation of blood vessels including the basal membrane, endothelial cell and adjacent alveolar structures as shown in **Figures 1**–**3**. Clinically, the suspicion of VEXAS syndrome was raised by the persistent systemic inflammation and the presence of cytopenias, which prompted the ILD board to suggest diagnostic testing for the VEXAS syndrome. Reassessment of bone marrow biopsy revealed vacuolization in granulocytic precursor cells (**Figure 4**) (15). Genetic testing of peripheral blood finally confirmed a somatic UBA1 (p.Met41.Val) mutation via next-generation sequencing (NGS).

## 3 Outcome and follow-up

Following diagnosis of VEXAS syndrome, treatment with ruxolitinib 20mg BID was initiated and step-wisely increased to 40 mg BID while prednisolone was slowly tapered to a low dose treatment less than 10mg. This led to initial clinical stabilization, reduction of CRP, resolution of fever, and improvement in fatigue and hematological parameters. During the further course, there appeared to be gradual progression of hematological abnormalities, despite intensified therapy. When ruxolitinib was abruptly discontinued by the patient’s family physician due to a COVID-19 infection, the patient witnessed respiratory deterioration with recurrence of bilateral pulmonary infiltrates and renal failure requiring intensive care treatment with dialysis. Clinical stabilization could again be achieved by high-dose hydrocortisone and antibiotic therapy.

Shortly after discharge on medium-dose steroids and ruxolitinib, the patient was readmitted with dizziness and mild ataxia. Brain MRI showed multi-embolic ischemic lesions suggestive of thromboembolic complications. Regular follow-up appointments with rheumatology, hematology and pneumology were continued. Phases of stability followed by new deteriorations given the relapsing nature of the disease. Long-term corticosteroid therapy became increasingly challenging, due to diabetic and vascular complications including a localized leg infection related to preexisting peripheral arterial disease that finally led to lower limb amputation. Due to the worsening of the cytopenias and increasing need for blood transfusions, an alternative steroid-sparing therapy next to ruxolitinib was being discussed, and tocilizumab 10 mg/kg body weight was initiated intravenously every four weeks. After two administrations, there was no improvement, with CRP levels remaining elevated and cytopenia persisting. The patient’s general state of health further declined which caused him to opt against another trial of a JAK inhibitor or other drugs in addition to prednisolone. He passed away at the institutional palliative care unit nine months after the diagnosis of VEXAS syndrome.

## 4 Discussion

This case illustrates the diagnostic and therapeutic challenges of VEXAS syndrome, especially during the early phases of the disease, when differential diagnoses such as IgG4-RD, idiopathic organizing pneumonia or systemic vasculitis may be present. The initial presentation with periorbital swelling and elevated serum IgG4 suggested IgG4-RD, however, the ACR/EULAR 2019 classification criteria were not fulfilled due to the lack of histopathological confirmation. Elevated IgG4 serum levels are a non-specific finding, and alternative diagnoses must be ruled out before a diagnosis of IgG4-RD can be confirmed (14). The ongoing systemic symptoms, and especially the cytopenia and PET/CT bone marrow uptake did not comply with IgG4-RD. Organizing pneumonia was suspected based on radiologic and histologic findings but is a non-specific manifestation of various diseases including autoimmune conditions and also VEXAS syndrome (5, 16). Cutaneous leukocytoclastic vasculitis had also been observed, but in the absence of ANCA and with uniform vacuolization in the bone marrow, systemic vasculitis was excluded. The differential diagnosis of VEXAS syndrome was brought up during multidisciplinary board discussion due to the syndromic pattern consisting of refractory inflammation, cytopenia, pulmonary involvement, bone marrow histology, hepatosplenomegaly, and subsequent genetic testing for UBA1 mutation. This is consistent with the current literature and ACR guidelines considering the importance of genetic testing in males over 50 years with unclear systemic inflammation and bone marrow vacuolization (5, 15–19). In a recent study by Baggio et al. (2024), the role of peripheral blood cytology in the diagnosis of VEXAS syndrome was highlighted. Specifically, the identification of vacuolization in peripheral blood cells can serve as an important diagnostic feature to distinguish from other inflammatory conditions, such as IgG4-RD (20).

Despite guidelines being available by now (6), treatment of VEXAS syndrome remains challenging. High-dose steroid therapy usually leads to a therapeutic response but is poorly tolerated on long-term (9). Our patient responded initially to prednisolone but relapsed again during corticosteroid tapering. The additional medication with ruxolitinib led to a temporary disease stabilization, which is in accordance with recent findings that JAK inhibitors may influence hyperinflammation in VEXAS (21). Considering the multi-embolic ischemic lesions the patient experienced, we initially suggested that they might be potentially related to disease activity and the abrupt termination of ruxolitinib therapy (22, 23). However, thrombosis in VEXAS syndrome is mainly described as venous thromboembolism (6). Additionally, the patient’s underlying atherosclerotic disease, including ischemic heart disease and several cardiovascular risk factors, JAK inhibitor treatment must also be considered as significant contributing factors. It was observed that while ruxolitinib was paused, a significant increase in CRP and progressive cytopenia occurred (24). This temporal correlation underlines the association with therapy, although a clear causal relationship has not yet been established due to the lack of controlled data (9, 21, 25). Other treatment options including, azacitidine or IL-6 inhibitors as stated in the ACR guidelines used on a case-to-case basis and also have been shown effective (1, 7, 26).

Recent case series highlight the challenges of persistent steroid dependence and frequent misclassification as vasculitis or IgG4-RD (3). Mortality remains high in some VEXAS phenotypes, especially those with hematological progression (5, 9). Further larger-scale, multicenter and prospective clinical trials are warranted to further assess the efficacy and safety of different existing or newly developed treatment options.

Ultrastructural changes through transmission electron microscopy (TEM) of lung tissue in VEXAS syndrome have not yet been described in the literature. In the field of interstitial lung diseases, transbronchial cryobiopsies have already been reported as feasible with similar image quality compared to surgical sample excision (27). In our case, TEM imaging of endothelial structures showed severe damage, with complete occlusion of the capillaries by these pseudopodia formation in some images (**Figure 1**). The basement membrane appeared thickened and fibrous, with massive amounts of underlying collagen visible (**Figure 2**). In the alveoli, electron-dense material that could not be clearly identified was present and could possibly be interpreted as phagosomes (**Figure 3**). There was no evidence of vacuolated myeloid cells in TEM imaging, and no additional testing for a UBA1 mutation was performed on lung tissue. Overall, these detected abnormalities most certainly reflect local tissue damage, possibly due to inflammation, and may limit gas exchange explaining patient’s deteriorating respiratory situation. However, it is unclear whether those findings are specific for VEXAS and if they were caused by disease-specific inflammatory mechanisms, or if they just reflect unspecific lung injury. Still, they may provide valuable insights into the pathophysiology of the lung manifestations of VEXAS syndrome and could stimulate future research in this field.

## 5 Learning points

- Consider VEXAS syndrome in older men with systemic inflammation and cytopenia, especially when work-up for alternative diagnoses such as IgG4-RD or vasculitis are inconclusive.
- Elevated serum IgG4 levels and organizing pneumonia may mimic IgG4-RD, but do neither allow a diagnosis of IgG4-rd nor do they exclude VEXAS. Bone marrow evaluation and genetic confirmation are essential.
- Evaluation of vascular, basal membrane, and alveolar alterations using transmission electron microscopy (TEM) from transbronchial lung cryobiopsies may offer valuable insight into pathophysiology of VEXAS syndrome.
- Multidisciplinary evaluation and early genetic diagnostics are crucial to avoid diagnostic delay and enable targeted treatment in complex inflammatory syndromes.

## Data Availability

The original contributions presented in the study are included in the article/**Supplementary Material**. Further inquiries can be directed to the corresponding author.
