# Correction: Case Report: Diagnostic challenges in VEXAS syndrome with novel ultrastructural lung findings: IgG4-RD and vasculitis as relevant differential diagnoses

**DOI:** 10.3389/fimmu.2026.1805595

**Published:** 2026-05-19

**Authors:** Peter Etzel, David Lang, Walter Stoiber, Astrid Obermayer, Georg Öberseder, Bernd Lamprecht

**Affiliations:** 1 Johannes Kepler University Linz, Kepler University Hospital, Department of Pulmonology, Linz, Austria; 2 Department of Environment and Biodiversity – EM Core Facility, University of Salzburg, Salzberg, Austria; 3 Kepler University Hospital, Department of Rheumatology, Linz, Austria

**Keywords:** organizing pneumonia, ruxolitinib, transbronchial lung cryobiopsy, transmission electron microscopy (TEM), UBA1 mutation

Authors Walter Stoiber and Astrid Obermayer were omitted as authors in the published article. The correct author list reads:

Peter Etzel1, David Lang1, Walter Stoiber2, Astrid Obermayer2, Georg Öberseder3, Bernd Lamprecht1

1 Johannes Kepler University Linz, Kepler University Hospital, Department of Pulmonology, Linz, Austria

2 Department of Environment and Biodiversity – EM Core Facility, University of Salzburg, Salzberg, Austria

3 Kepler University Hospital, Department of Rheumatology, Linz, Austria

The Author Contributions Statement has been corrected to read:

PE: Writing – review & editing, Writing – original draft. DL: Writing – review & editing. WS: Writing – review & editing, Investigation, Resources, Visualization. AO: Writing – review & editing, Investigation, Resources, Visualization. GÖ: Writing – review & editing. BL: Resources, Writing – review & editing, Supervision.

There was a mistake in the caption of **Figure 1** as published. The corrected caption of **Figure 1** appears below.

“Ultrastructure of alveolar wall (arrow) in TBLC as seen in TEM, showing a pneumocyte with unclear electron-dense inclusions that could be interpreted as phagosomes (asterisk).”

There was a mistake in the caption of **Figure 2** as published. The corrected caption of **Figure 2** appears below:

“TEM of TBLC showing a massively thickened basement membrane with abundant collagen (asterisk) underneath a capillary with an endothelium of partly stratified appearance (arrows).”

There was a mistake in the caption of **Figure 3** as published. The corrected caption of **Figure 3** appears below:

“TEM image of lung tissue from a TBLC. Capillaries (arrow) may be almost completely blocked by pseudopodia. The structure marked with an asterisk is most likely an endothelial cell nucleus with aberrant indentations probably resulting from shear stress.”

The original version of this article has been updated.

