# Supplementary figures and images for: Case Report: Diagnostic challenges in VEXAS syndrome with novel ultrastructural lung findings: IgG4-RD and vasculitis as relevant differential diagnoses
Source: Front Immunol. 2026 Jan 21;16:1742328. doi: 10.3389/fimmu.2025.1742328 (PMC12867857; doi:10.3389/fimmu.2025.1742328)

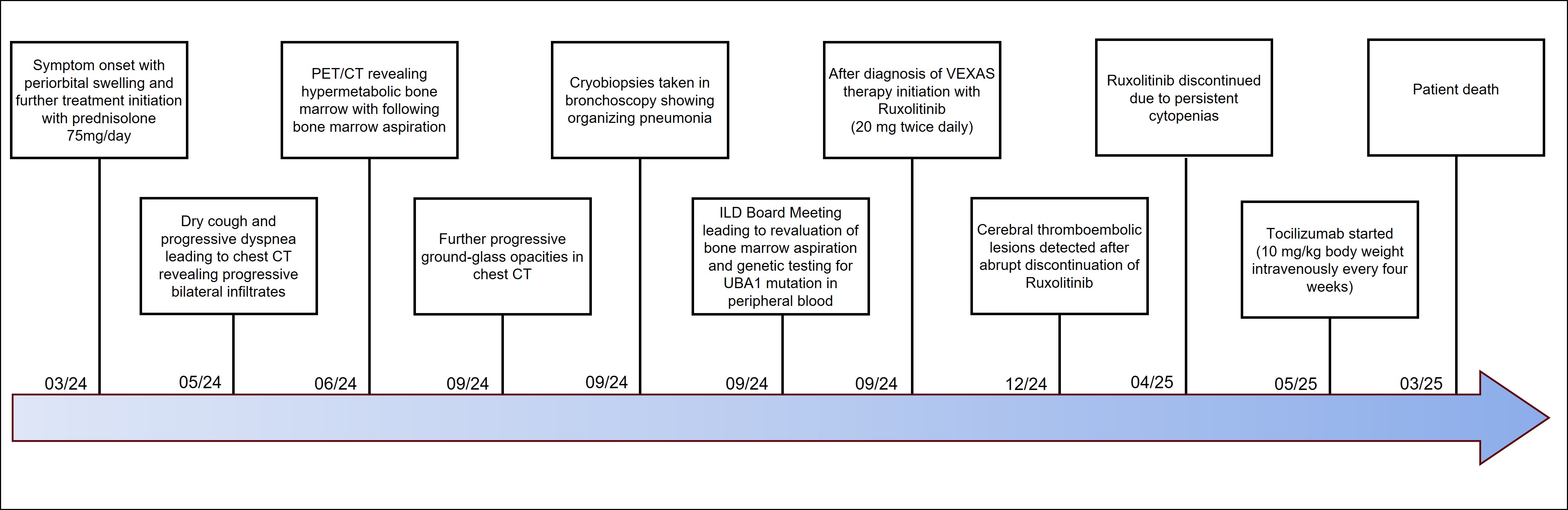

Supplement: Supplementary file 1 [file Image1.jpeg]
